# *Listeria monocytogenes* Infection Causes Metabolic Shifts in *Drosophila melanogaster*

**DOI:** 10.1371/journal.pone.0050679

**Published:** 2012-12-13

**Authors:** Moria C. Chambers, Kyung Han Song, David S. Schneider

**Affiliations:** Department of Microbiology and Immunology, Stanford University, Stanford, California, United States of America; University of Illinois at Chicago College of Medicine, United States of America

## Abstract

Immunity and metabolism are intimately linked; manipulating metabolism, either through diet or genetics, has the power to alter survival during infection. However, despite metabolism's powerful ability to alter the course of infections, little is known about what being “sick” means metabolically. Here we describe the metabolic changes occurring in a model system when *Listeria monocytogenes* causes a lethal infection in *Drosophila melanogaster*. *L. monocytogenes* infection alters energy metabolism; the flies gradually lose both of their energy stores, triglycerides and glycogen, and show decreases in both intermediate metabolites and enzyme message for the two main energy pathways, beta-oxidation and glycolysis. *L. monocytogenes* infection also causes enzymatic reduction in the anti-oxidant uric acid, and knocking out the enzyme uric oxidase has a complicated effect on immunity. Free amino acid levels also change during infection, including a drop in tyrosine levels which may be due to robust *L. monocytogenes* induced melanization.

## Introduction

We need to be careful with the way we draw lines around biological systems. For example, survival during infection is affected by most physiologies in the body and thus it seems dangerous to gather one group of proteins that are responsible for killing microbes and say “these define immunology.” It matters little if we can explain “the immune system” but don't understand all that helps us survive infections. *Drosophila* are a useful innate immunity model, but are also useful for studying microbial pathogenesis to find these “non-immune” factors. Metabolism is an example of such a system that influences immunity and, in *Drosophila*, the only explanations we have for how microbes cause disease involve changes in energy stores or gut function [1]–[4]. Though we don't have a broad understanding of what defines a “sick” metabolic state is during an infection several experiments in Drosophila focus on select groups of metabolites.

Research in *Drosophila melanogaster* has repeatedly ascribed metabolism a role in immunity. *Mycobacterium marinum* is a model pathogen that causes “consumption” in flies; infected flies lose all of their energy stores and waste to death. Reduction of FoxO, a negative regulatory transcription factor in the insulin signaling pathway, slows the infection induced wasting and increases the mean time to death [1]. FoxO is also important in modulating the transcript levels of some immune effectors, anti-microbial peptides (AMPs). In the absence of an infection, starvation causes a FoxO-dependent upregulation of AMPs, effective even in the absence of Toll and Imd, the most notable regulators of AMP transcription [5], [6]. Additionally, FoxO binds directly to the promoter of Drosomycin, suggesting that these are direct effects of the protein. Taken together, these data support an intimate connection between insulin and immune signaling.

The relationship between metabolism and immunity is complex and the results depend upon the pathogen tested and measured immune output. Ayres and colleagues found that *Listeria monocytogenes* and *Salmonella typhimurium* cause infection-induced anorexia. In return, diet restriction alters a fly's tolerance and resistance to infection in a pathogen specific manner, making it worse at resisting *L. monocytogenes* but better able to tolerate *S. typhimurium* [7]. *Drosophila* larvae fed *Pseudomonas entomophila* have a melanized proventriculus and consume less food, which is potentially one cause of death from infection [3], [4]. Pathology also may occur because *P. entomophila* causes gut damage using a pore-forming protein, monalysin [2].

Work in other insects supports the link between diet composition and immunity; in caterpillars of the African spotted leaf worm (*Spodoptera littoralis*), Cotter and colleagues showed that the protein and carbohydrate content of food impacted the animals' immune responses in a pathway dependent manner and that there were potential trade-offs and no “perfect” immune diet [8]. Adamo and colleagues showed that infected crickets (*Gryllus texensis*) were less resistant to infection when fed high fat diets and would self-select lower fat diets when infected and given a choice [9]. These studies not only show that a beneficial diet is infection dependent but that animals possess the ability to distinguish and choose more beneficial diets when infected.

Drosophila C Virus infection of the *Drosophila* S2 cell line revealed a specific role for a single metabolic pathway: fatty acid biosynthesis [10]. RNAi knockdown of components of the synthetic pathway inhibited viral replication. More recently this has been extended into mammalian systems showing that regulation of fatty acid biosynthesis by AMPK is important for a number of viruses [11]. This does not appear to be solely a viral pathogenesis phenomenon as branched chain fatty acids promote intracellular growth of *Listeria monocytogenes* in a mammalian cell line [12]. The ability to obtain energy is important to both host and bacterium and changes in metabolism and nutrition likely impacts both. While each of these papers highlights a narrow aspect of the metabolism-immunity relationship, a more global approach to metabolite changes has the benefit of revealing system-wide changes and unanticipated roles for metabolites.

Here, we take a broad look at the changes in metabolism seen during *L. monocytogenes* infection of *Drosophila*. We chose *L. monocytogenes* as a pathogen because it causes infection-induced anorexia and a previously published microarray analysis from the lab showed infection-induced transcriptional changes in a number of metabolic pathways [7], [13]. By combining both a metabolic focused analysis of our microarray to look at metabolism transcripts and broad spectrum chemistry approach to look at the metabolites themselves, a picture emerges of a shifting energy landscape in infected fruit flies.

*L. monocytogenes* infection reduces metabolite concentrations in the two primary energy generation pathways: beta-oxidation and glycolysis. The messages encoding enzymes in these pathways are also down regulated. As the infection proceeds, triglyceride and glycogen stores fall. In addition, two shifts in metabolites were related to reactive oxygen species (ROS) generation. First, there was enzymatically driven decrease in the anti-oxidant, uric acid, which has a complicated effect on immunity. Second, there was a decrease in tyrosine, an important precursor for L-3,4-dihydroxyphenylalanine (L-DOPA), the substrate for phenol oxidase, which generates microbe killing ROS and helps resolve infections.

## Results

### Infection with *L. monocytogenes* affects a broad range of metabolites

**Figure 1:**
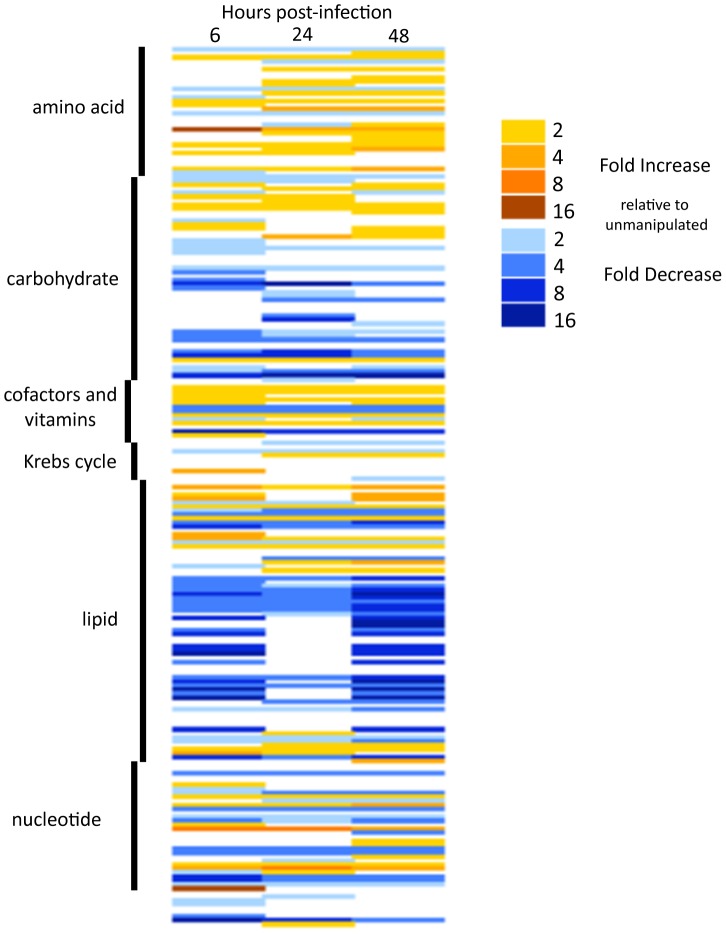
*L. monocytogenes* infection affects many metabolites. Flies infected with *L. monocytogenes* were analyzed by Metabolon using GC-MS and LC-MS to measure metabolites that changed during infection as determined by the Welch two sample t-test. This is a heat map generated based on the fold change observed, grouped by metabolic pathway. White rectangles indicate no significant changes relative to unmanipulated.

We measured changes in metabolite concentrations at 0, 6, 24, and 48 hours post-infection and found that 169 metabolites, out of the 221 identified, were significantly different during at least one time-point after infection (Figure 1 and Supplementary Figures 1–5). These metabolites fell into the following broad categories: amino acid metabolism (Supplementary Figure 1), carbohydrate metabolism (Supplementary Figure 2), lipid metabolism (Supplementary Figure 3), nucleotide metabolism (Supplementary Figure 4), and additional miscellaneous categories (Supplementary Figure 5).

### *L. monocytogenes* infection affects two major energy pathways: beta-oxidation and glycolysis

Past work from our group demonstrated that flies infected with *M. marinum* suffered from a wasting disease that reduced the two main energy stores, fat (triglycerides) and glycogen [1]. Transcript levels data from our *L. monocytogenes* microarray suggested that transcripts for enzymes in the beta-oxidation and glycolysis pathways were similarly down regulated, indicating that energy metabolism might be altered during this infection as well.

**Figure 2:**
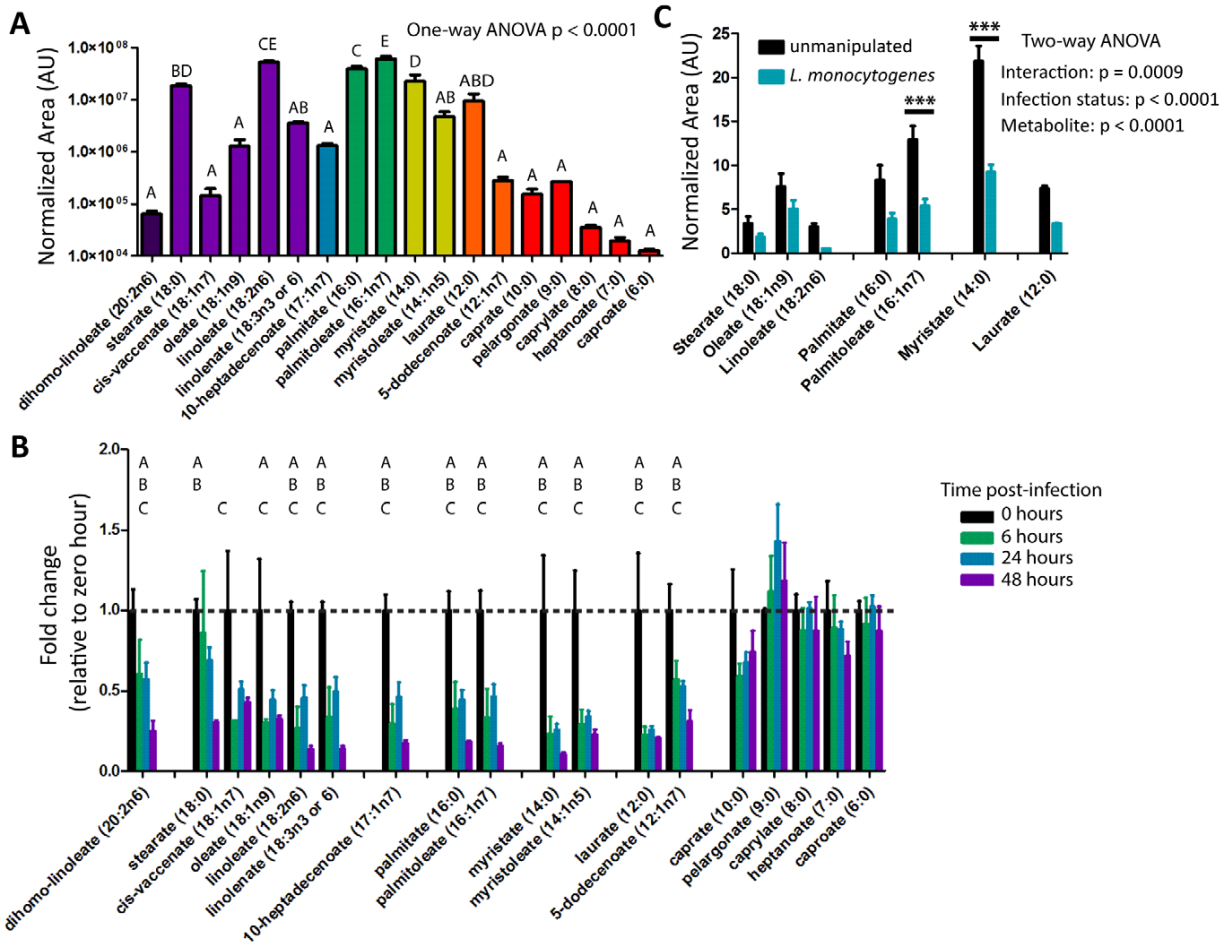
Long-chain fatty acids decrease during *L. monocytogenes* infection. (A–B) Levels of medium and long chain fatty acids as extracted from metabolon data set. (A) Fatty Acid levels of uninfected flies. The Normalized Area was determined by taking the area under each GC/LC peak divided by area under the standard peak, metabolite levels compared by One Way ANOVA and grouped by Tukey Test (q = 0.05) (B) Fatty Acid levels during *L. monocytogenes* infection, values normalized to zero hour for each compound. Significantly different values determined by Welch's two-tailed t-test (p<0.05; 0 hr vs 48 hr: A, 0 hr vs 24 hr: B, 0 hr vs 6 hr: C) (C) Levels of Long Chain fatty acids during infection as confirmed by independent GC-MS. Normalized Area determined by taking the area under each GC peak divided by area under the standard peak. Statistical significance was determined by ANOVA with Bonferroni post-test for individual metabolites (* p<0.05, *** p<0.001).

**Figure 3:**
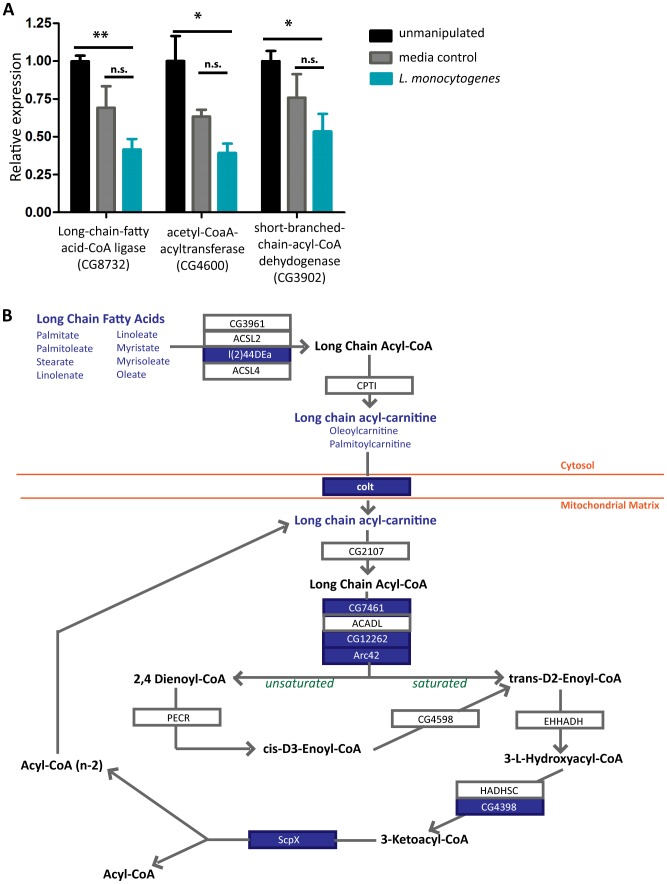
Changes in beta-oxidation during *L. monocytogenes* infection. (A) Expression levels of three genes involved in beta-oxidation (CG8732, CG4600, CG3902) six hours after *L. monocytogenes* infection as assayed by qRT-PCR. Expression levels for each gene were normalized to the level of transcript in unmanipulated flies. Significance was determined by a one-tailed t-test (* p<0.05, ** p<0.01). (B) Metabolite and gene changes within beta-oxidation during *L. monocytogenes* infection. Genes selected in Genespring 12.0 using Oneway ANOVA with Welch's correction for unequal variance (p<0.05) are highlighted in blue boxes. Metabolites that are significantly down during infection as determined by a Welch's two-tailed t-test (* p<0.05) are in blue type.

We first examined how fat metabolism shifted during *L. monocytogenes* infection. Triglycerides are composed of three fatty acids joined by ester bonds connecting their carboxyl groups to the hydroxyl groups of glycerol. Triglycerides are broken down by lipases which release free fatty acids and these are then transported into the mitochondria where they are metabolized through beta-oxidation. The basal levels of free fatty acids in uninfected flies varied depending on both length of the carbon chain and saturation state (Figure 2A). Upon infection, all free fatty acids with twelve or more carbons decreased regardless of initial levels or saturation state (Figure 2B). We confirmed the *L. monocytogenes* induced decrease in fatty acids by specifically isolating free fatty acids and running in-house gas chromatography–mass spectrometry (GC-MS) (Figure 2C). We also confirmed the reduction in message for three genes (CG8732 – long chain fatty acid-CoA ligase, CG4600 – acetyl-CoA acyltransferase, CG3902 – short-branched-chain-acyl-CoA dehydrogenase) implicated by our previously published microarray by qRT-PCR (Figure 3A). Seven out of the 17 transcripts assayed by microarray decreased, although this does not include the rate limiting enzyme acyl-carnitine transferase (CPT-1: CG12891). The changes in beta-oxidation are summarized in Figure 3B.

**Figure 4:**
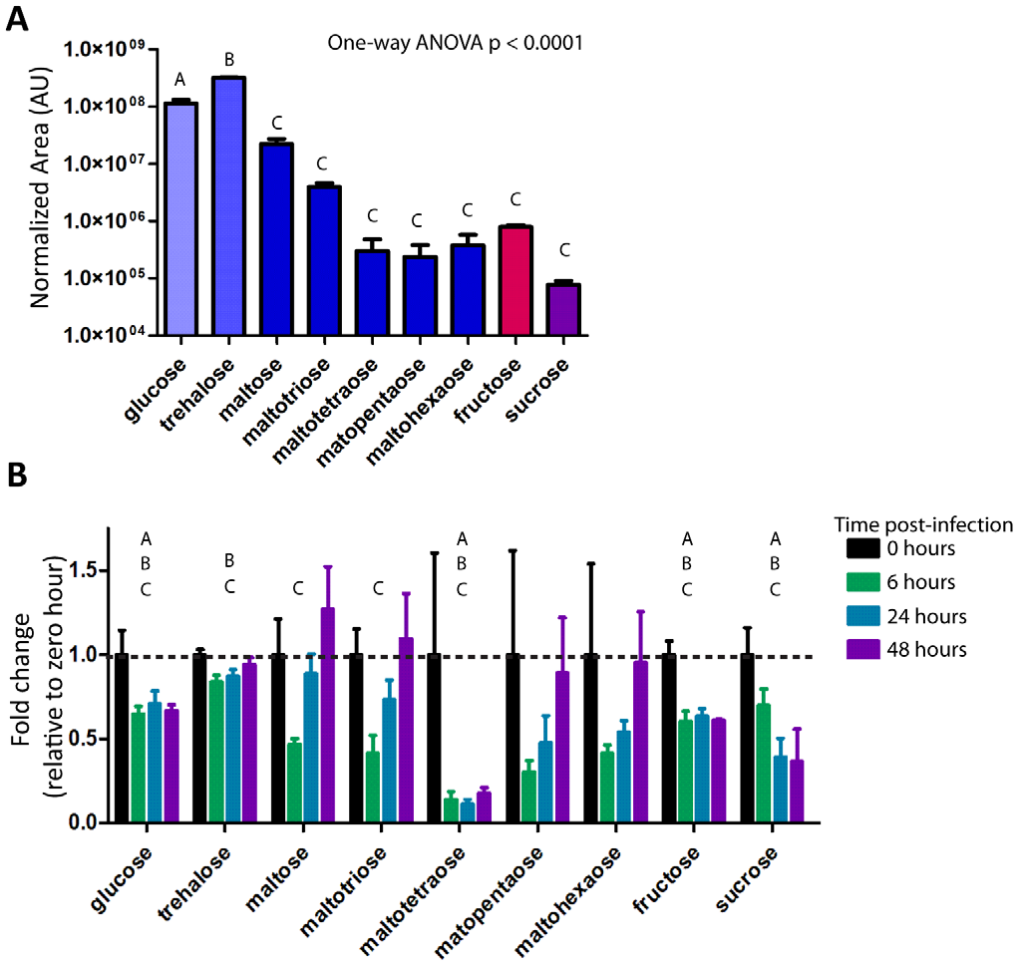
Select simple and complex sugars decrease during *L. monocytogenes* infection. Simple and complex sugar levels as extracted from metabolon data set. (A) Sugar levels in uninfected flies. The Normalized Area was determined by taking the area under each GC/LC peak divided by area under the standard peak, metabolite levels compared by One Way ANOVA and grouped by Tukey Test (q = 0.05). (B) Sugar levels during *L. monocytogenes* infection, values normalized to zero hour for each compound. Significantly different values determined by Welch's two-tailed t-test (p<0.05; 0 hr vs 48 hr: A, 0 hr vs 24 hr: B, 0 hr vs 6 hr: C).

**Figure 5:**
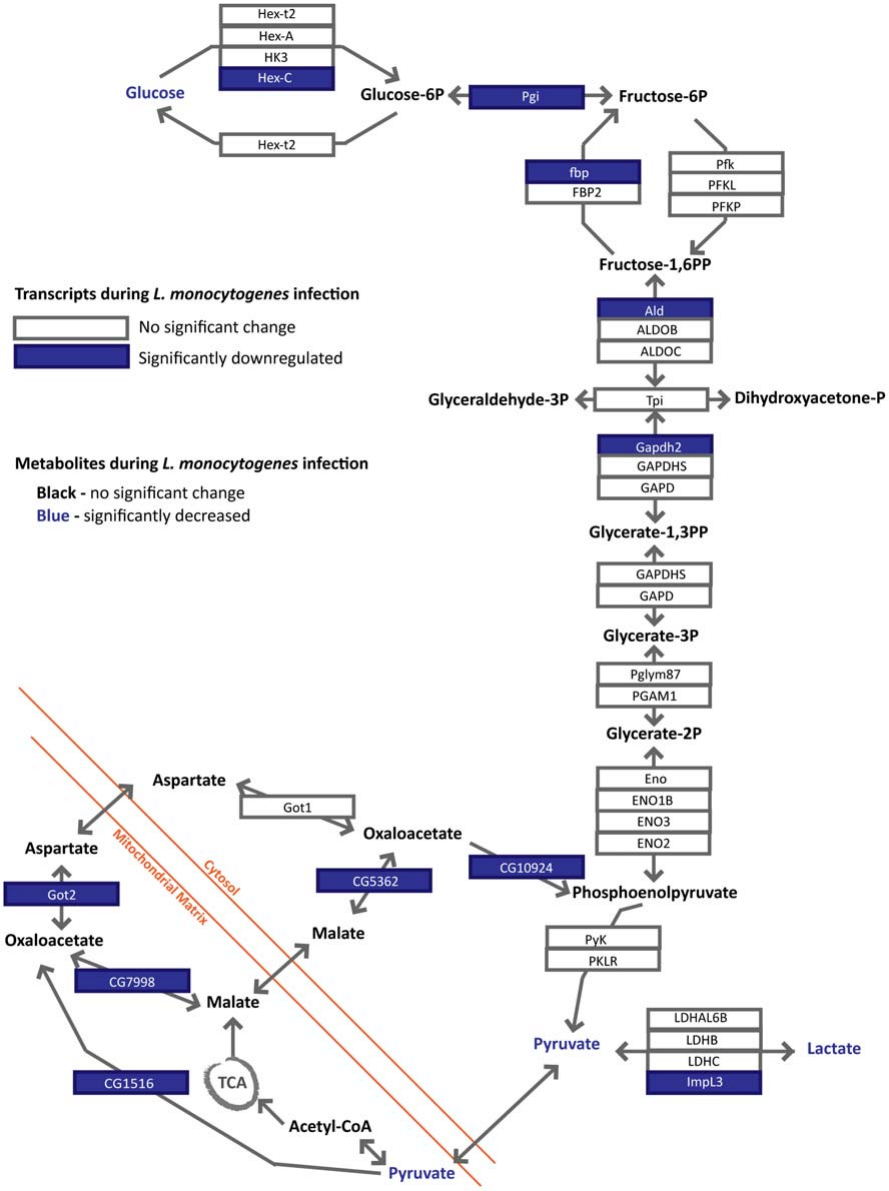
Summary of changes in glycolysis during *L. monocytogenes* infection. Metabolite and gene changes within glycolysis during *L. monocytogenes* infection. Genes selected in Genespring 12.0 using Oneway ANOVA with Welch's correction for unequal variance (p<0.05) are highlighted in blue boxes. Metabolites that are significantly down during infection as determined by a Welch's two-tailed t-test (* p<0.05) are in blue type.

Glycogen and sugar metabolism during *L. monocytogenes* infection presents a slightly more complicated picture. Glycogen is broken down by glycogen phosphorylase into Glucose-1-P, which is a substrate for glycolysis. Glucose and other more complex sugars can also enter into glycolysis. During infection with *L. monocytogenes*, the sugars can be broken into three groups, those with no significant change, those with an intermittent shift and those that drop and stay low. The primary circulatory sugar in flies, trehalose, drops for the first days of infection but rises back to baseline by 48 hours post-infection (Figure 4B). Glucose, fructose, sucrose and maltotetraose, a complex sugar composed of four glucose molecules, all remain low during infection (Figure 4B). The dimers of glucose, maltose and trehalose, along with maltotriose, a glucose trimer, decrease intermittently during infection (Figure 4B). The compounds that do not significantly shift are the longer glucose complexes, maltopentaose and maltohexaose, which are present at relatively low levels in the fly and show higher variation (Figure 4B). Eleven out of thirty eight transcripts assayed by microarray decreased; and although the rate limiting enzyme for glycolysis phopho-fructokinase (Pfk: CG4001) does not decrease significantly, the rate limiting enzyme for gluconeogenesis, fructose-1,6-bisphosphatase (fbp: CG31692), does. Figure 5 summarizes the transcriptional and metabolite effects of *L. monocytogenes* on glycolysis.

### *L. monocytogenes* infection decreases energy stores

**Figure 6:**
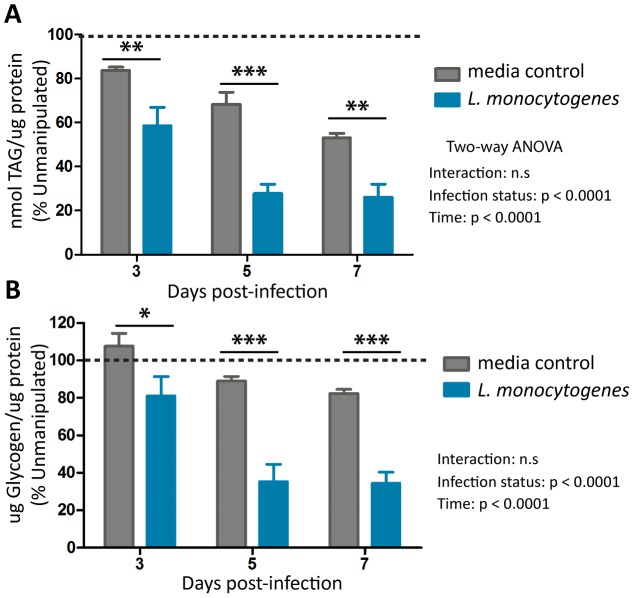
*L. monocytogenes* infection reduces energy stores in *D. melanogaster*. Triglyceride (A) and glycogen (B) levels were assayed during later time-points of *L. monocytogenes* infection. Metabolite levels were normalized to total protein and then represented as percent of the levels in unmanipulated flies. The significant sources of variation were assessed by two-way ANOVA and differences in the metabolite levels at each time point were assessed by the Bonferroni post-test after ANOVA; significantly different values denoted by asterisk (* p<0.05, ** p<0.01, *** p<0.001).

To assess the long-term effect these rapid changes in beta-oxidation and glycolysis had on the energy storage in infected flies, we measured triglyceride and glycogen levels during infection. We found that flies infected with *L. monocytogenes* have significantly reduced levels of both triglycerides (Figure 6A) and glycogen (Figure 6B). This indicates an overall decrease in energy stores.

### A potent anti-oxidant, uric acid, is enzymatically reduced during infection

Uric acid is a potent anti-oxidant that is present in human blood at near saturating concentrations and is considered to provide over half of the blood's anti-oxidant power [14]. In the fly, uric acid is produced as a waste product of purine elimination, and accumulates in malpighian tubules, filling them with uric acid crystals, which are then dumped into the hind gut where they are excreted in the frass [15]. Flies encode the enzyme uricase, which converts uric acid into the more soluble allantoin. This reaction is highly conserved in the animal kingdom although it is notably missing in higher apes (including humans) and Dalmatians [16], [17]. Uric acid can also be converted to allantoin via a reactive oxygen driven chemical reaction, which can happen in all animals. This is why, in humans, allantoin is a biomarker for reactive oxygen (ROS) stress as it can only be made as the product of reactive oxygen species and uric acid [18].

**Figure 7:**
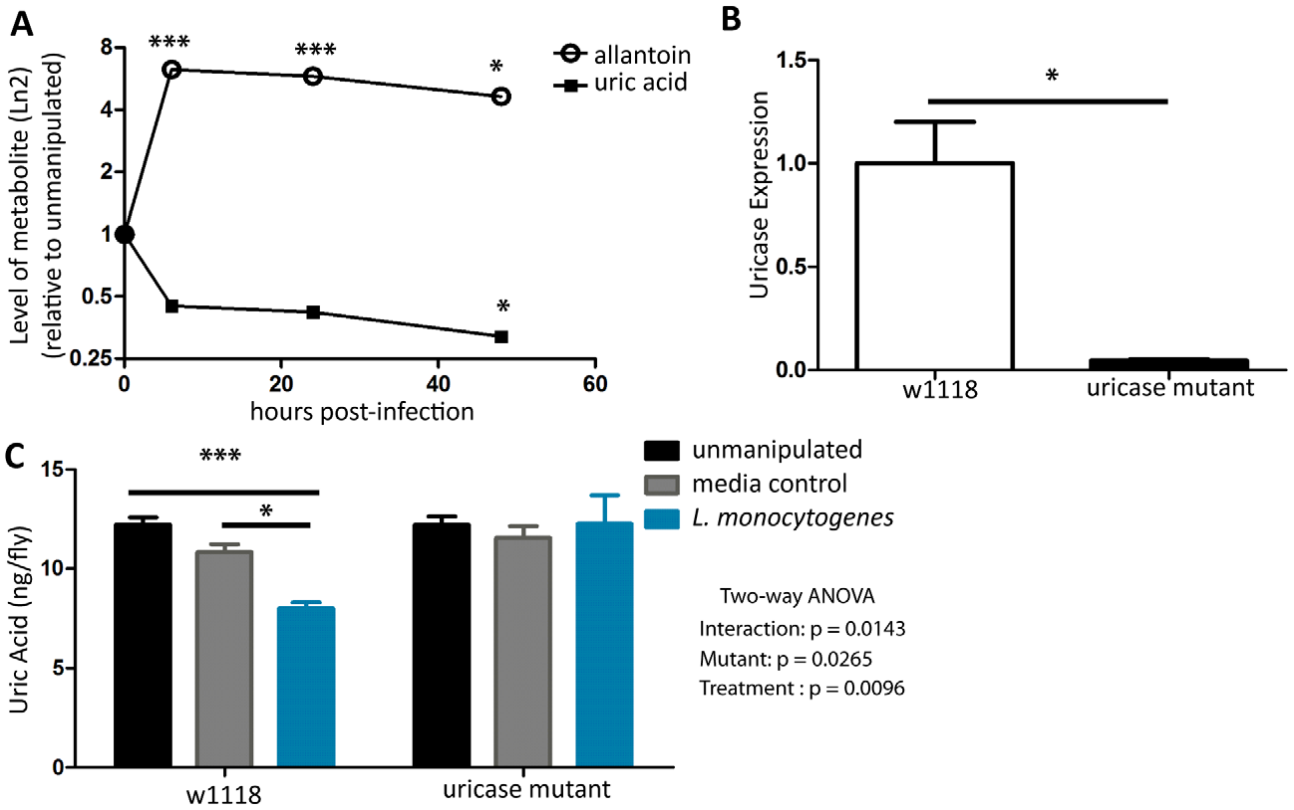
Uricase causes a drop in uric acid during *L. monocytogenes* infection. (A) Uric acid and allantoin levels as extracted from metabolon data set, significance was determined by a Welch's two-tailed t-test (* p<0.05, *** p<0.0001) (B) qRT-PCR for uricase expression in unmanipulated flies, significance determined by a Welch's two-tailed t-test (* p<0.05). (C) Uric acid levels after injection with either media or *L. monocytogenes*. The significant sources of variation were assessed by two-way ANOVA and differences in bacterial load between fly lines at each time point were assessed by the Bonferroni post-test after ANOVA and significantly different values denoted by asterisk (* p<0.05, *** p<0.001).

During *L. monocytogenes* infection of *D. melanogaster* the levels of uric acid decrease and allantoin its metabolic product increase (Figure 7A). Why does this happen? Is it the result of enzymatic breakdown or an indication that ROS is being produced? Uricase is upregulated 3-fold during *L. monocytogenes* infection supporting a role for the enzymatic pathway (Chambers MC, 2012). To test the role of uricase in modulating uric acid levels we looked at the effect of infection on uric acid levels in a uricase mutant. We expected that if the uric acid decrease was primarily driven by the ROS and uric acid reaction then the mutant would still have an infection induced decrease in uric acid. However, a uricase mutant, which has a 10-fold knockdown of uricase message (Figure 7B), had no change in uric acid levels during infection. This supports the idea that uricase is the primary effector of the infection-induced decreases in uric acid levels.

### Uricase has a complicated affect on immunity

**Figure 8:**
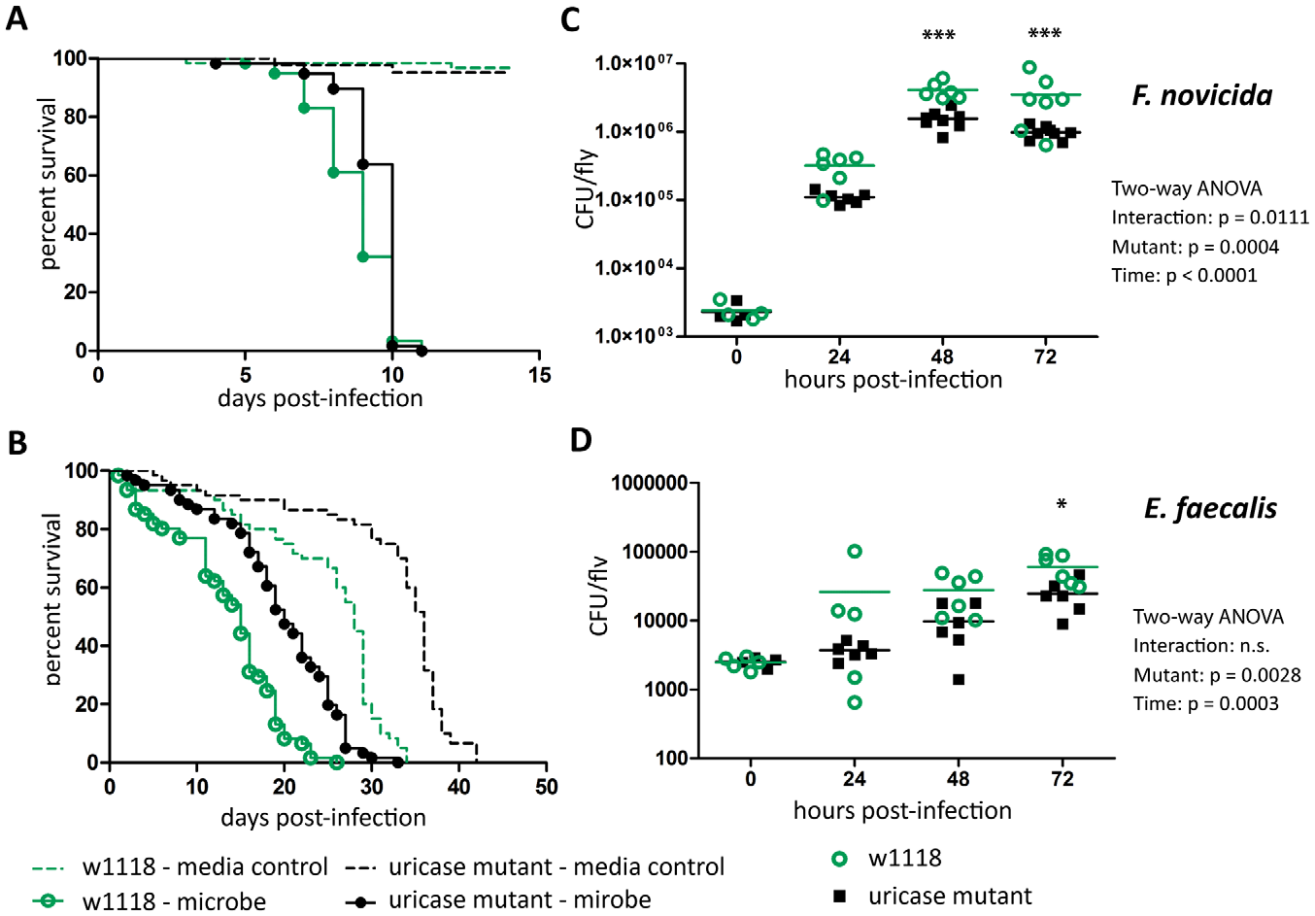
Uricase mutants have increased resistance to *F. novicida* and *E. faecalis*. Flies were injected and monitored for survival (A) *F. novicida* (B) *E. faecalis*. Log-rank analysis of the survival curves gives p<0.0001 (w/o media controls in analysis). Flies were injected and CFUs monitored at various time points post injection (C) *F. novicida* (G) *E. faecalis*. The significant sources of variation were assessed by two-way ANOVA and differences in bacterial load between fly lines at each time point were assessed by the Bonferroni post-test after ANOVA and significantly different values denoted by asterisk (* p<0.05, *** p<0.001).

**Figure 9:**
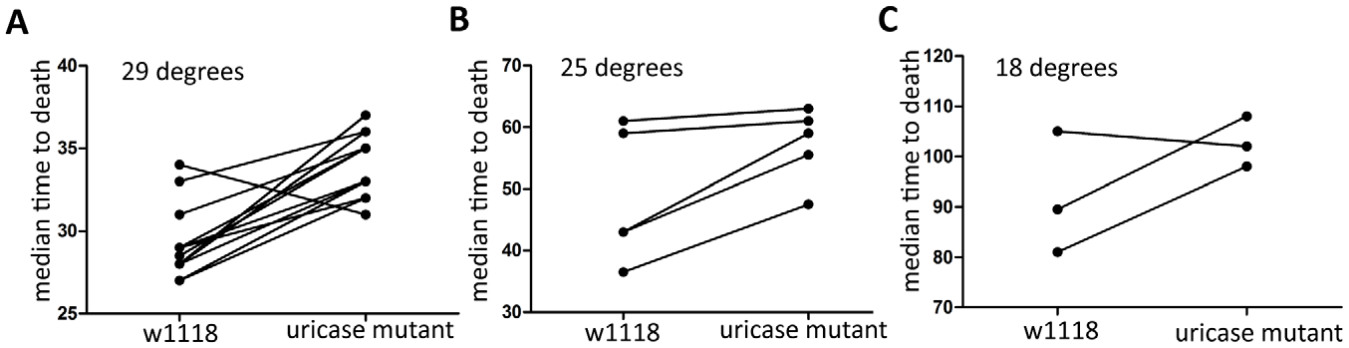
Uricase mutants have increased survival during wounding. Flies were injected with media and monitored for survival at (A) 29°C (B) 25°C (C) 18°C. Significance was determine by paired t-test (29°C, p<0.0001; 25°C, p = 0.0381;18°C, p = 0.2584).

We next wanted to assay how uric acid regulation would affect the host during infection. Why would the host alter a potential antioxidant when it was going to produce ROS? The immune implications of uricase regulation are complicated. Uricase mutants do not have a consistent significant change in survival or bacterial load during *L. monocytogenes*, *S. typhimurium*, and *Burkholderia cepacia* infection (data not shown). Uricase mutants are, however, more resistant to *Francisella novicida* (Figure 8A,C) and *Enterococcus faecalis* (Figure 8B,D) infection. The uricase mutants also better survive wounding as seen in Figure 9. Supplementary Figure 2 shows the summary of all media controls and shows a significant increase in median time to death at both 29°C and 25°C (p<0.0001 and p = 0.0381 respectively). The lifespan of unmanipulated flies, however, is unchanged relative to the parental line (data not shown).

### Amino acid pre-cursors for melanization decrease during infection

**Figure 10:**
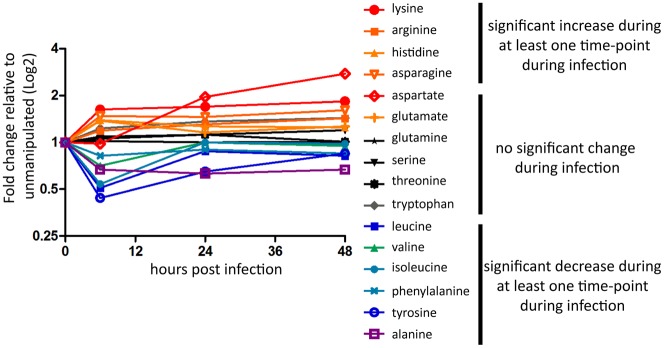
Free amino acid levels shift during *L. monocytogenes* infection. Amino acid levels as extracted from metabolon data set, significance determined by a Welch's two-tailed t-test (* p<0.05), warm colors indicate a significantly higher level during at least one time-point post infection, cool colors indicate a significantly lower level during at least one time-point post infection, gray colors indicate no significant changes.

Melanization is an important part of the insect immune response and is required for resistance to both *L. monocytogenes* and *S. typhimurium* which both cause robust disseminated melanization in the fly. The primary enzyme responsible for melanization, phenoloxidase (PO) uses L-3,4-dihydroxyphenylalanine (L-DOPA) and this is synthesized by a pathway which converts phenylalanine to tyrosine to L-DOPA. We find that both phenylalanine and tyrosine are depleted through infection and that L-DOPA levels are also down at 6 hours post-infection (Figure 10, Supplementary Figure 1). This is in contrast to many other amino acids, which remain constant or even increase during infection.

## Discussion

Immunity is tightly linked to metabolism, and this paper discusses a range of connections, from anti-oxidants to energy stores, at both the transcript and metabolite level. The changes are likely a product of integrating host energy needs, changes in feeding during infection, energy requirements of the bacteria, and virulence driven changes in host biology.

Most prominent in our data was the impact that *L. monocytogenes* infection has on fruit fly energy metabolism. Similar to what was seen in *M. marinum* infected flies, these *L. monocytogenes* infected flies also suffered a consumption-like disease with decreases in both triglyceride and glycogen levels [1]. Preceding these decreases in energy stores, the flies showed a marked decrease in the immediate substrates for mitochondrial beta-oxidation and glycolysis. Concomitantly, the flies also had decreased investment in the transcripts for both of these pathways. The entire energy landscape of these flies appears to be in flux. There are many potential functional causes and consequences of these changes. These flies are diet restricted and potentially increasing energy expenditure to mount an immune response; these are both host factors which could drain energy reserves. On the bacterial side, *L. monocytogenes* is replicating during infection potentially consuming resources as well. One could imagine the host shifting its metabolism to reduce available nutrient for the bacteria, further impacting these pathways. There is likely a complicated interplay between all of these factors, and manipulating any of these components has the potential to impact the survival of the host. It could be a multi-faceted trade-off between host nutrition, bacterial nutrition and investment in immunity.

Another potential trade-off is the enzymatic reduction in the anti-oxidant uric acid and this also has a compelling potential host-bacteria interaction story. Theoretically, keeping the level of an anti-oxidant high during infection could impede the effectiveness of the ROS immune effector response because it could protect the infecting bacteria as well as the host. Antioxidants would thus allow the bacteria to grow to higher levels, but also improve tolerance to infection. However, we found that stabilization of uric acid levels had no effect during a number of infections and the flies better resisted the bacteria during *F. novicida* and *E. faecalis* infection. This is the opposite of what we expected. We have two potential explanations.

First, changes in uric acid metabolism could lead to compensating changes in the levels of other anti-oxidants. Second, uric acid could have an additional role in immune or metabolic signaling that is yet unappreciated. This story is further complicated by effect of wounding on uricase mutants; loss of uricase increases survival during wounding and this makes it difficult to deconstruct the immune phenotype.

During infection, we also saw that amino acid precursors for melanization, a core immune response against *L. monocytogenes* decreased. This decrease could be due to the robust *L. monocytogenes* induced melanization response; however, bacteria can import amino acids through permeases and transporters, and shifts in the free amino acid composition could also be due to bacterial use.

These metabolic data provide a stepping stone from which we can launch additional and more targeted studies on metabolism. Studies of metabolic-immune interactions could potentiate development of cheaper treatments for infection for if we can modulate our resistance and tolerance through diet shift instead of expensive pharmaceuticals, we have the potential to bring treatment to a much larger swath of the global community.

## Materials and Methods

### Fly Stocks

The wild-type parental stain used in all experiments is white^1118^ (Bloomington 6326). Uricase experiments were done using a piggybac mutant (Bloomington 18814).

### Bacterial Strains and Culture Conditions

*Listeria monocytogenes* (strain 10403S) cultures were grown in 4 ml brain heart infusion (BHI) broth at 37°C without shaking after inoculation from *L. monocytogenes* grown overnight on a Luria Bertani (LB) agar plate. *L. monocytogenes* stocks were stored at −80°C in BHI broth containing 15% glycerol.

*Francisella novicida* (strain U1112) cultures were grown in 4 ml tryptic soy broth (TSB) supplemented with 0.2% L-cysteine at 37°C on a rotating wheel after inoculation from *F. novicida* stocks stored at −80°C in TSB broth containing 15% glycerol.

*Enterococcus faecalis* (strain V583) cultures were grown in 4 ml LB broth at 37°C with shaking after inoculation from *E. faecalis* grown overnight on a LB agar plate. *E. faecalis* stocks were stored at −80°C in BHI broth containing 15% glycerol.

For infection, 50 nL of the bacterial cultures were injected at the following optical densities (OD_600_): *L. monocytogenes*, 0.01 (approx. 1,000 CFU/fly); *F. novicida*, 0.001 (approx. 1,000 CFU/fly); *E. faecalis*, 0.6 (approx. 5,000 CFU/fly).

### Injection

Five to seven day post-eclosion male flies were used for injection. The flies were raised at 25°C, 65% humidity on yeasted dextrose food in a light cycling incubator (12 hours dark, 12 hours light). Flies were anesthetized with CO_2_. A picospritzer (Parker Hanninfin, http://www.parker.com) was used to inject 50 nL of liquid into each fly with pulled glass capillary needles that were individually calibrated by measuring the size of the expelled drop under oil. About 20 flies were placed per vial and then experiments were kept at 29°C, 65% humidity in a light cycling incubator.

### Metabolomics

Flies were injected with 50 nL of *L. monocytogenes* culture (about 1000 CFU/fly) or left unmanipulated. Following injection, the flies were placed in dextrose vials and incubated at 29°C. At 0, 24, 48 and 72 hours post-injection triplicates of 100 flies were flash frozen and then homogenized in methanol. These preparations were then sent to Metabolon (http://www.metabolon.com), which performed a combination of Gas and Liquid Chromotography techniques combined with Mass Spectrometry (GC/LC-MS). A table of metabolites that changed in our samples was returned both with the raw normalized area for each metabolic peak and with calculated amounts relative to zero hour w1118 flies. Statistical significant differences were determined using Welch's two tailed t-test.

### Fatty-Acid Isolation and Quantification

Isolation and quantification protocols closely followed Nandakumar M et al. [19]. Briefly, 10 flies were homogenized in 300 uL of 3% H_2_SO_4_. The solution and accompanying fly debris were then transferred to glass tubes and the total volume brought to 4 mL (with 3% H_2_SO_4_). Tubes were capped and incubated at 80°C for two hours. After allowing samples to cool, 1.5 mL water and 750 uL of hexane plus standard (I-Br decane) were added. Each sample was vortexed and then spun at 550 rpm for about one minute without brake. Take 200–400 uL of the top layer and apply to GC-MS.

### Quantitative RT-PCR

Flies were injected with 50 nL of *L. monocytogenes* OD_600_ = 0.01 (about 1000 CFU/fly) or kept unmanipulated. Following injection, the flies were placed in dextrose vials and incubated at 29°C for six hours. Groups of 12 flies were homogenized in TriZOL and stored at −80°C until processed. RNA was isolated using a standard TriZOL preparation, and the samples were treated with DNase (Promega, http://www.promega.com). Quantitative RT-PCR was performed as described previously by Schneider et al. using a Bio-Rad icycler and the following primer sets: CG8732, CG4600, CG3902, and uricase (Supplementary Table 1) [20]. Briefly, relative levels of mRNA were determined using a standard curve and then normalized to 15a expression levels. Samples were then normalized to the unmanipulated w1118 expression level to yield fold induction.

### Triglyceride and Glycogen Quantification

Quantification of triglyceride and glycogen levels was assayed using kits from BioVision. The protocol provided by the manufacturer was followed. Samples were prepared by homogenizing 5 flies in 125 uL of the assay buffer Tris-EDTA (TE; 10 mM Tris, 1 mM EDTA, pH 8) with 0.1% Triton X-100. All samples were normalized to total protein content, values are represented as a percent relative to unmanipulated flies.

### Uric Acid Quantification

Quantification of uric acid levels was done using a kit from BioVision. The protocol provided by the manufacturer was followed. Samples were prepared by homogenizing 5 flies in 200 uL of the assay buffer.

### Survival Experiments

Mutant flies and the parental control were injected with 50 nL of the bacterial culture or medium at the following optical densities (OD_600_): *L. monocytogenes*, 0.01 (approx. 1,000 CFU/fly); *F. novicida*, 0.001 (approx. 1,000 CFU/fly); *E. faecalis*, 0.6 (approx. 5,000 CFU/fly).. About sixty flies were assayed for each condition and placed in three vials of 20 flies each. Death was recorded daily. Survival curves are plotted as Kaplan-Meier plots and statistical significance is tested using log-rank analysis using Prism software (http://www.prism-software.com). All experiments were performed at least three times and yielded similar results.

### CFU determination

Colony forming units (CFUs) were determined using both spot-plating and an autoplate spiral plater (Spiral Biotech http://www.aicompanies.com). For spot-plating, eight individual flies were collected at each time point. These flies were homogenized, diluted serially and plated onto the appropriate media (LB agar for *L. monocytogenes*/*E. faecalis* and Mueller-Hinton agar supplemented with ferric pyrophosphate (0.05%) and L-cysteine (0.1%) for *F. novicida*) and grown overnight at 37°C. Some *L. monocytogenes* experiments were completed using the Spiral Biotech plater and for these six individual flies were homogenized and diluted. 50 µL of liquid was plated exponentially on a LB plate, grown overnight at 37°C and then counted using QCount, which calculates the original number of CFU per fly. For statistical analysis, if CFU/fly did not approximate a Gaussian distribution we analyzed the log(10) transform of the data. CFU experiments were assessed for sources of variation using a two-way ANOVA and followed with Bonferroni post-tests for specific comparisons of interest.

## Supporting Information

Figure S1 **Amino Acid Pathway Metabolites.** Metabolites assayed by a combination of GC/LC-MS. All values are presented as a fold change relative to uninfected flies. Significantly increased metabolites, as determined by a Welch's two-tailed T-Test, are in red cells, and significantly decreased metabolites are in blue cells.
(TIF)

Figure S2 **Carbohydrate Pathway Metabolites.** Metabolites assayed by a combination of GC/LC-MS. All values are presented as a fold change relative to uninfected flies. Significantly increased metabolites, as determined by a Welch's two-tailed T-Test, are in red cells, and significantly decreased metabolites are in blue cells.
(TIF)

Figure S3 **Lipid Pathway Metabolites.** Metabolites assayed by a combination of GC/LC-MS. All values are presented as a fold change relative to uninfected flies. Significantly increased metabolites, as determined by a Welch's two-tailed T-Test, are in red cells, and significantly decreased metabolites are in blue cells.
(TIF)

Figure S4 **Nucleotide Pathway Metabolites.** Metabolites assayed by a combination of GC/LC-MS. All values are presented as a fold change relative to uninfected flies. Significantly increased metabolites, as determined by a Welch's two-tailed T-Test, are in red cells, and significantly decreased metabolites are in blue cells.
(TIF)

Figure S5 **Miscellaneous Pathway Metabolites.** Metabolites assayed by a combination of GC/LC-MS. All values are presented as a fold change relative to uninfected flies. Significantly increased metabolites, as determined by a Welch's two-tailed T-Test, are in red cells, and significantly decreased metabolites are in blue cells.
(TIF)

Table S1 **qRT-PCR Primers.** Sequences of both the forward and reverse primers used for the qRT-PCR experiments.
(DOCX)

## References

[1] Dionne MS, Pham LN, Shirasu-Hiza M, Schneider DS (2006) Akt and FOXO dysregulation contribute to infection-induced wasting in Drosophila. Curr Biol 16: 1977–1985. 17055976 10.1016/j.cub.2006.08.052

[2] Opota O, Vallet-Gely I, Vincentelli R, Kellenberger C, Iacovache I, et al (2011) Monalysin, a novel ss-pore-forming toxin from the Drosophila pathogen Pseudomonas entomophila, contributes to host intestinal damage and lethality. PLoS Pathog 7: e1002259. 21980286 10.1371/journal.ppat.1002259 PMC3182943

[3] Liehl P, Blight M, Vodovar N, Boccard F, Lemaitre B (2006) Prevalence of local immune response against oral infection in a Drosophila/Pseudomonas infection model. PLoS Pathog 2: e56. 16789834 10.1371/journal.ppat.0020056 PMC1475658

[4] Vodovar N, Vinals M, Liehl P, Basset A, Degrouard J, et al (2005) Drosophila host defense after oral infection by an entomopathogenic Pseudomonas species. Proc Natl Acad Sci U S A 102: 11414–11419. 16061818 10.1073/pnas.0502240102 PMC1183552

[5] Becker T, Loch G, Beyer M, Zinke I, Aschenbrenner AC, et al (2010) FOXO-dependent regulation of innate immune homeostasis. Nature 463: 369–373. 20090753 10.1038/nature08698

[6] Brown AE, Baumbach J, Cook PE, Ligoxygakis P (2009) Short-term starvation of immune deficient Drosophila improves survival to gram-negative bacterial infections. PLoS One 4: e4490. 19221590 10.1371/journal.pone.0004490 PMC2637427

[7] Ayres JS, Schneider DS (2009) The role of anorexia in resistance and tolerance to infections in Drosophila. PLoS Biol 7: e1000150. 19597539 10.1371/journal.pbio.1000150 PMC2701602

[8] Cotter SC, Simpson SJ, Raubenheimer D, Wilson K (2011) Macronutrient balance mediates trade-offs between immune function and life history traits. Functional Ecology 25: 186–198.

[9] Adamo SA, Bartlett A, Le J, Spencer N, Sullivan K (2010) Illness-induced anorexia may reduce trade-offs between digestion and immune function. Animal Behaviour 79: 3–10.

[10] Cherry S, Kunte A, Wang H, Coyne C, Rawson RB, et al (2006) COPI activity coupled with fatty acid biosynthesis is required for viral replication. PLoS Pathog 2: e102. 17040126 10.1371/journal.ppat.0020102 PMC1599761

[11] Moser TS, D S, Cherry S (2012) AMP-Activated Kinase Restricts Rift Valley Fever Virus Infection by Inhibiting Fatty Acid Synthesis. PLoS Pathog 8: e1002661. 22532801 10.1371/journal.ppat.1002661 PMC3330235

[12] Sun Y, O'Riordan MX (2010) Branched-chain fatty acids promote Listeria monocytogenes intracellular infection and virulence. Infect Immun 78: 4667–4673. 20823206 10.1128/IAI.00546-10 PMC2976352

[13] Chambers MC, Lightfield KL, Schneider DS (2012) How the Fly Balances Its Ability to Combat Different Pathogens. PLoS Pathog (accepted). 10.1371/journal.ppat.1002970 PMC3521699 23271964

[14] Ames BN, Cathcart R, Schwiers E, Hochstein P (1981) Uric acid provides an antioxidant defense in humans against oxidant- and radical-caused aging and cancer: a hypothesis. Proc Natl Acad Sci U S A 78: 6858–6862. 6947260 10.1073/pnas.78.11.6858 PMC349151

[15] Dow JA, Romero MF (2010) Drosophila provides rapid modeling of renal development, function, and disease. Am J Physiol Renal Physiol 299: F1237–1244. 20926630 10.1152/ajprenal.00521.2010 PMC3006309

[16] Wu XW, Lee CC, Muzny DM, Caskey CT (1989) Urate oxidase: primary structure and evolutionary implications. Proc Natl Acad Sci U S A 86: 9412–9416. 2594778 10.1073/pnas.86.23.9412 PMC298506

[17] Wu XW, Muzny DM, Lee CC, Caskey CT (1992) Two independent mutational events in the loss of urate oxidase during hominoid evolution. J Mol Evol 34: 78–84. 1556746 10.1007/BF00163854

[18] Il'yasova D, Spasojevic I, Wang F, Tolun AA, Base K, et al (2010) Urinary biomarkers of oxidative status in a clinical model of oxidative assault. Cancer Epidemiol Biomarkers Prev 19: 1506–1510. 20501773 10.1158/1055-9965.EPI-10-0211 PMC2883001

[19] Nandakumar M, Tan MW (2008) Gamma-linolenic and stearidonic acids are required for basal immunity in Caenorhabditis elegans through their effects on p38 MAP kinase activity. PLoS Genet 4: e1000273. 19023415 10.1371/journal.pgen.1000273 PMC2581601

[20] Schneider D, Shahabuddin M (2000) Malaria parasite development in a Drosophila model. Science 288: 2376–2379. 10875925 10.1126/science.288.5475.2376

