# Supplementary figures and images for: Listeria monocytogenes Infection Causes Metabolic Shifts in Drosophila melanogaster
Source: PLoS One. 2012 Dec 13;7(12):e50679. doi: 10.1371/journal.pone.0050679 (PMC3521769; doi:10.1371/journal.pone.0050679)

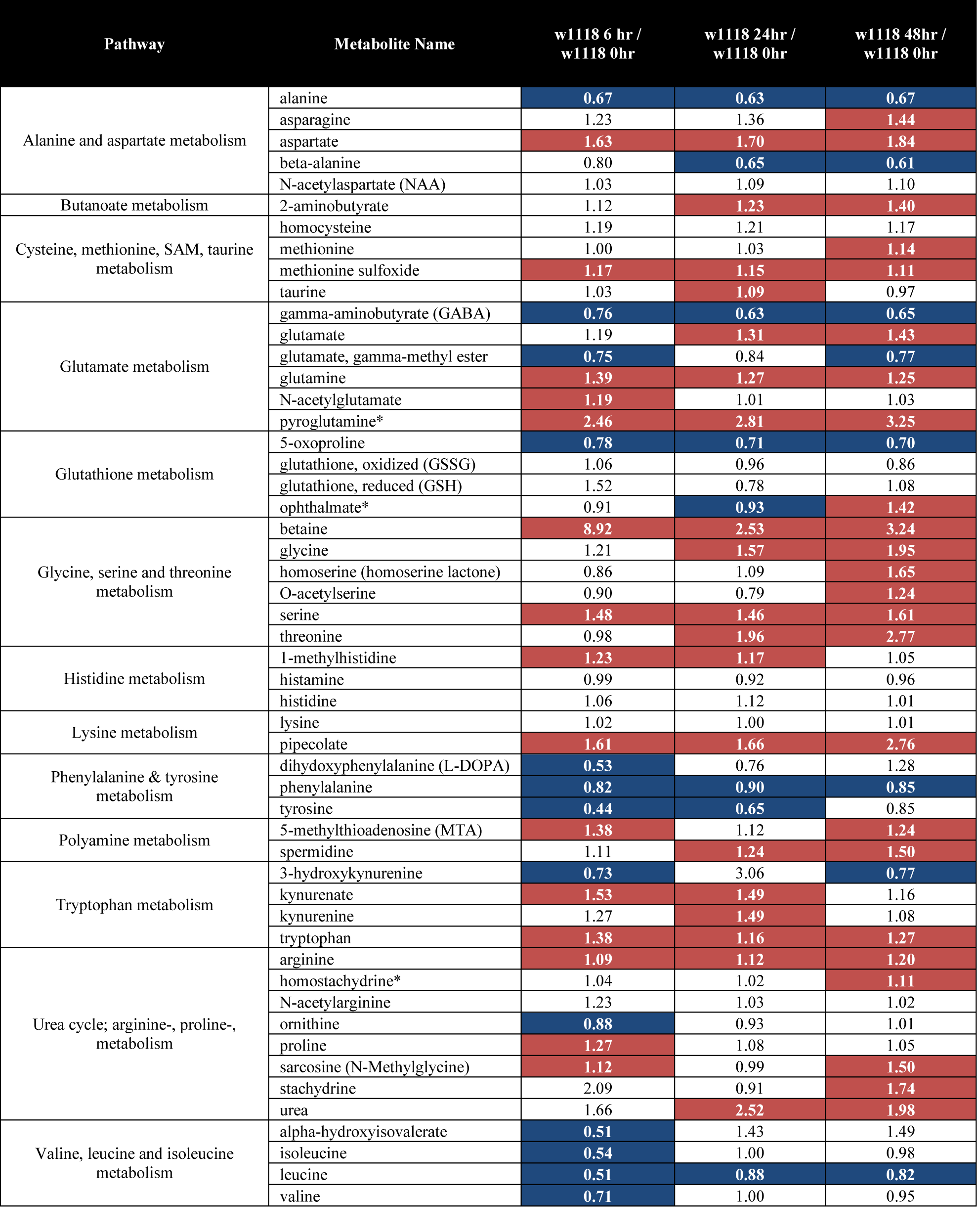

Supplement: Figure S1 — Amino Acid Pathway Metabolites. Metabolites assayed by a combination of GC/LC-MS. All values are presented as a fold change relative to uninfected flies. Significantly increased metabolites, as determined by a Welch's two-tailed T-Test, are in red cells, and significantly decreased metabolites are in blue cells. (TIF) [file pone.0050679.s001.tif]

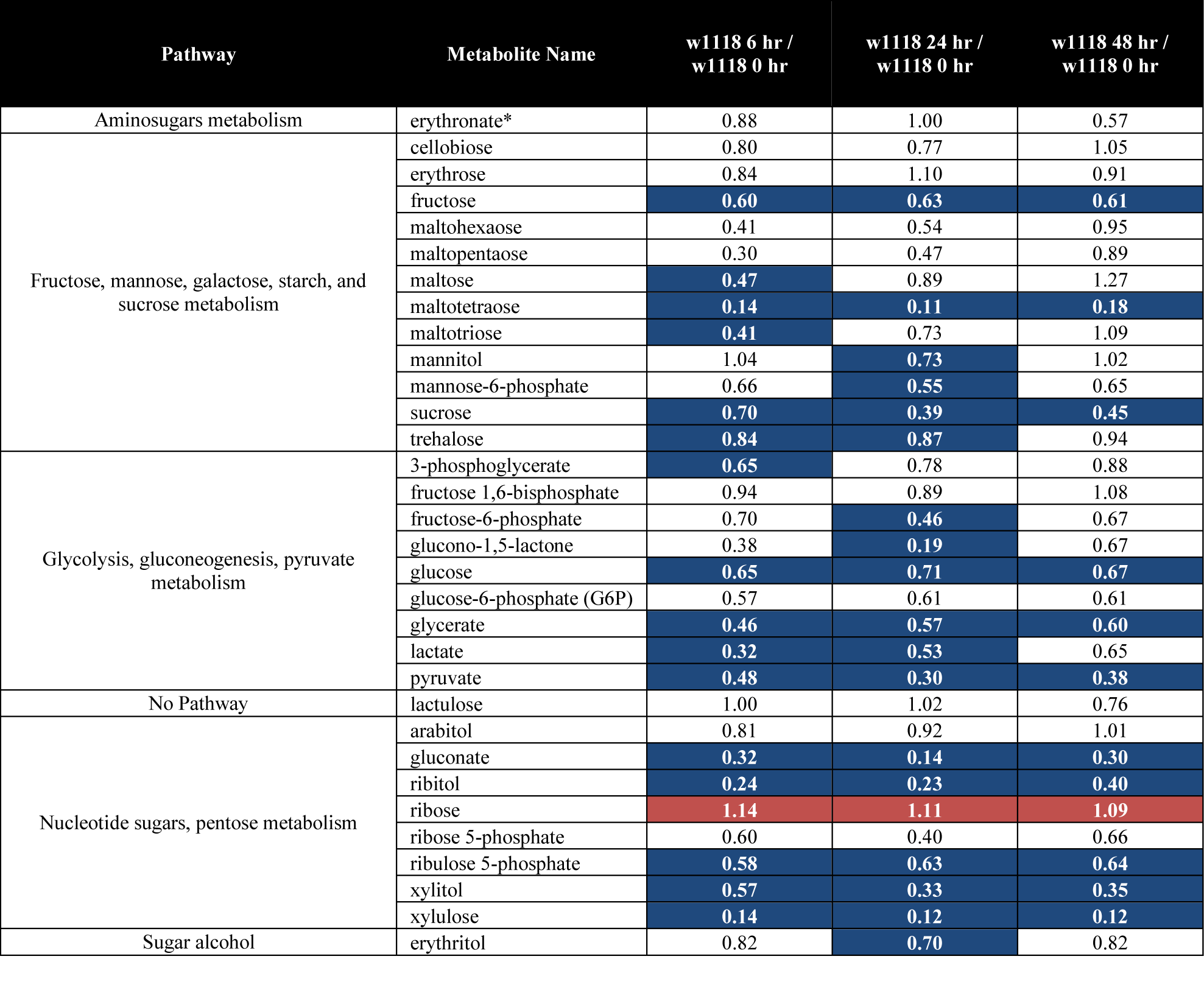

Supplement: Figure S2 — Carbohydrate Pathway Metabolites. Metabolites assayed by a combination of GC/LC-MS. All values are presented as a fold change relative to uninfected flies. Significantly increased metabolites, as determined by a Welch's two-tailed T-Test, are in red cells, and significantly decreased metabolites are in blue cells. (TIF) [file pone.0050679.s002.tif]

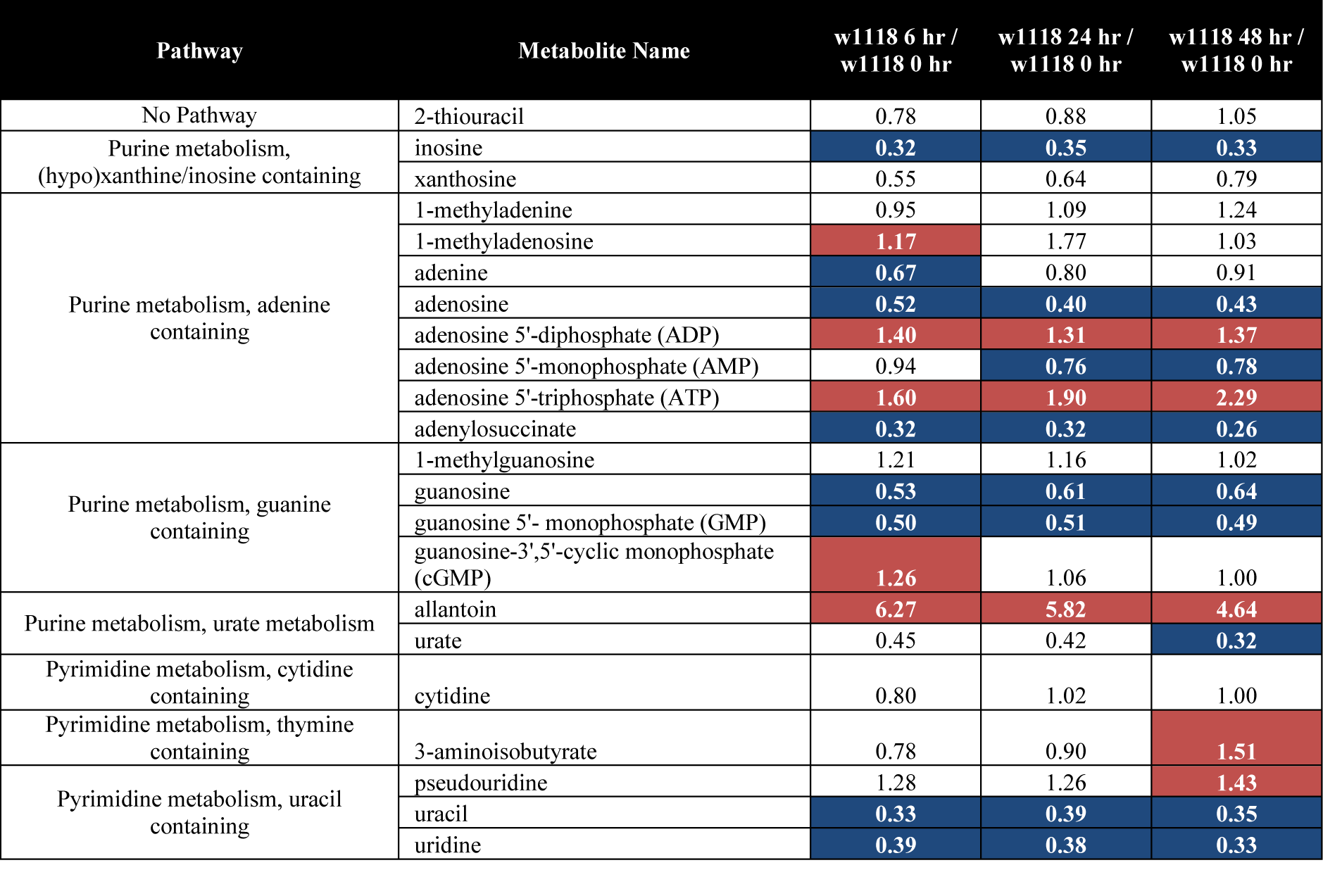

Supplement: Figure S4 — Nucleotide Pathway Metabolites. Metabolites assayed by a combination of GC/LC-MS. All values are presented as a fold change relative to uninfected flies. Significantly increased metabolites, as determined by a Welch's two-tailed T-Test, are in red cells, and significantly decreased metabolites are in blue cells. (TIF) [file pone.0050679.s004.tif]

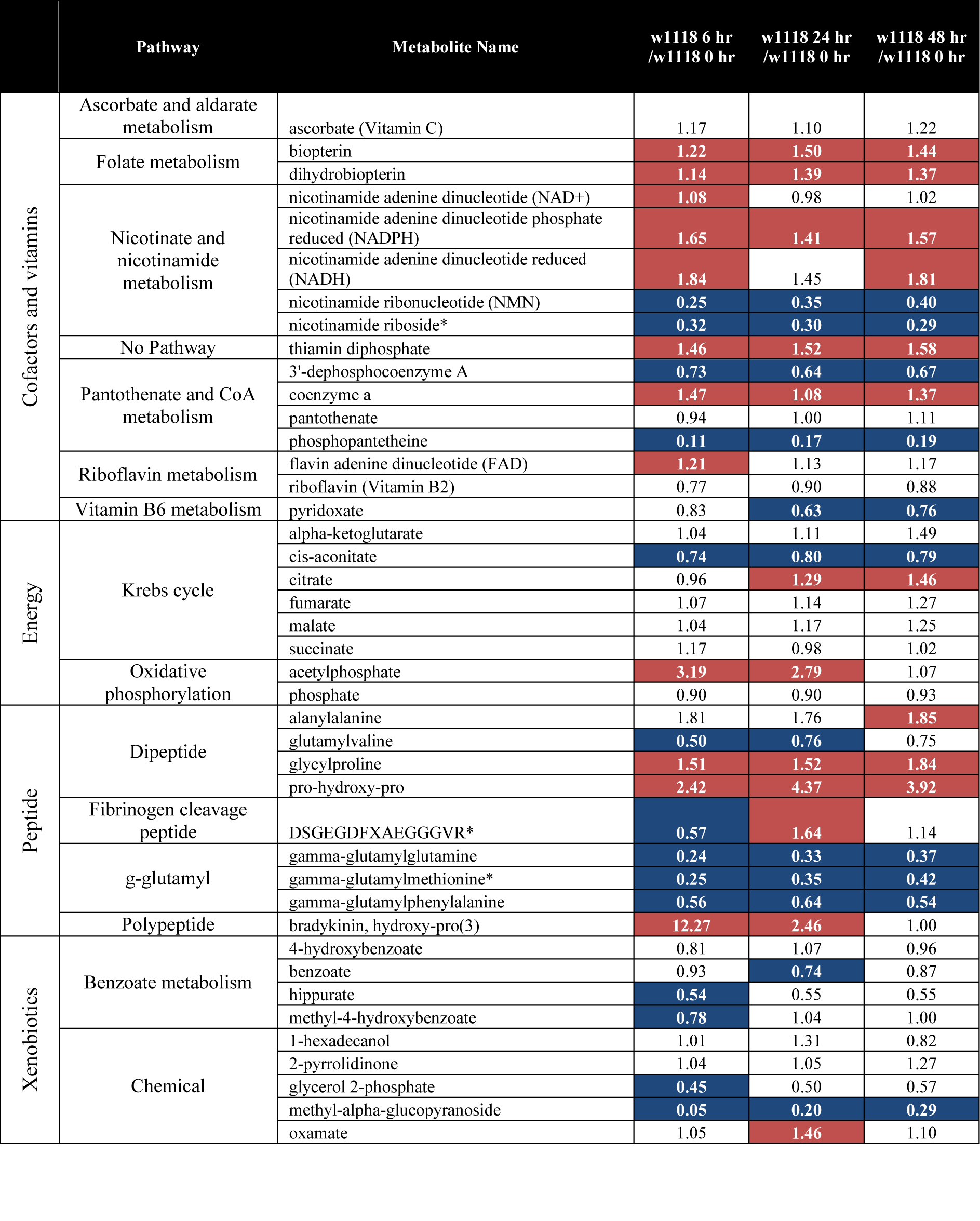

Supplement: Figure S5 — Miscellaneous Pathway Metabolites. Metabolites assayed by a combination of GC/LC-MS. All values are presented as a fold change relative to uninfected flies. Significantly increased metabolites, as determined by a Welch's two-tailed T-Test, are in red cells, and significantly decreased metabolites are in blue cells. (TIF) [file pone.0050679.s005.tif]
