# Supplementary material for: Listeria monocytogenes Infection Causes Metabolic Shifts in Drosophila melanogaster
Source: PLoS One. 2012 Dec 13;7(12):e50679. doi: 10.1371/journal.pone.0050679 (PMC3521769; doi:10.1371/journal.pone.0050679)
Supplement: Table S1 — qRT-PCR Primers. Sequences of both the forward and reverse primers used for the qRT-PCR experiments. (DOCX) [file pone.0050679.s006.docx]

**Table S1.** qRT-PCR primer sequences.

| Gene | CG Number | Forward Primer | Reverse Primer |
| --- | --- | --- | --- |
| Long-chain-fatty acid coA ligase | CG8732 | GAG AAC TTG GCC AGA AAC CA | GTG GGC AGC TAT CAT CCA TT |
| Acetyl-CoA-acyltransferase | CG4600 | GAG ATC AAC GAA GCC TTT GC | GCC TCC GTT CAC ATT CAG TT |
| Short-brached chain-acyl-CoA dehydrogenase | CG3902 | ACG TAA TCA ACG GCT CCA AG | AGG TGG TAA TGC CAC GGT AG |
| Uricase | CG7171 | GGG CAC TCA CCT GAA GTT GT | GGA CTT TCA ATG CCA TGC TT |
